# The Technic and Balancing of Local Anesthetics

**Published:** 1915-04

**Authors:** Josef Novitzky

**Affiliations:** San Francisco, Cal.


### THE TECHNIC AND BALANCING OF LOCAL ANESTHETICS.

BY JOSEF NOVITZKY, SAN FRANCISCO, CAL.

We have all been greatly interested in the new method of inducing local anesthesia by anesthetizing or blocking the nerve trunks supplying certain arêas. Many operations in surgery that required a general anesthetic can now be handled very nicely, and with less danger to the patient, by regional anesthesia. From many sides, however, comes the complaint of post-operative annoyance and irritation following these injections. My methods may not appear to differ from those in ordinary use; but as I have failed to observe any irritation or swelling that could be laid to the injection, possibly a discussion of the technic, preparation and use of solutions would be profitable. We must understand, however, that we are as yet only on the threshold of this work; and that up to 1901 and 1905—the dates on which adrenalin and novocain were given to science—nerve blocking with any amount of success was out of the question. The laws of osmosis, as applied to injections into the human body, are also in their infancy; and, according to the father of local anesthesia—Heinrich Braun—the application of these laws to our injection anesthetics will open new fields for medical research in the near future. By osmosis we mean the diffusion of fluids through membranes. The normal human blood freezes at —0.55 degrees. Any fluid that freezes at the same temperature is considered isotonic to human blood; that is, when it is injected into the human tissues it circulates through the body cells without change or injury to the cell. Its osmotic pressure is the same as that of the body fluids. If the injected fluid is weaker in osmotic pressure, we call it hypotonic; if stronger, it is hypertonic. Beyond certain limits both cause changes in the injected area. Taking 0.9 per cent. salt solution as normal or isotonic we find that all solutions below 0.6 per cent. cause marked swelling with tumefaction anesthesia. The hypertonic solutions, those above one per cent., cause a shrinking of the body cell through dehydration with localized paralysis. The more the solutions vary in saturation from normal, and in their freezing point from that of blood, the greater the osmotic change in the tissues. Plain sterile water will cause injection pain followed by swelling and anesthesia. According to Braun, when injected into a nerve sheath it causes instant anesthesia with permanent damage to its structures. Tumefaction, as well as dehydration, influences the action of the sensory nerves, and injures the tissues, irrespective of the substance in solution. Schleich, Braun and many others have demonstrated that a warm solution of normal salt injected under the skin will cause no injection pain, no anesthesia or swelling, and rapid absorption takes place with a restoration to normal. It follows, then, that if our anesthetic solutions are balanced or normal, we should secure an anesthesia with no after-effects; leaving the body structures in perfect condition.

The effect of a small amount of adrenalin—1-50,000 or 1-100,000—on the osmotic balance of novocain salt solution would be practically nil. In larger amounts there is considerable doubt as to its liability to act as an irritant directly on the cell envelope. The molecule of adrenalin is rather large, and such molecules do not lend themselves very well to osmotic circulation. It is thought that adrenalin is broken up in the tissues, and vessel walls, and probably absorbed by the lymphatic spaces. Adrenalin is also a constituent of the blood stream; and, in great dilution, is quite harmless as regards irritant action. The use of the synthetic suprarenalin tablets is strongly recommended because of their being free from any preservative to keep them stable and the fact that they keep indefinitely. Their tolerance to boiling is better than that of the organic form, and the possibility of making fresh solutions just before use lessens the danger of contamination. They are put up by the Faberwerke Hoechst people in tablets of one milligram (1-60 gr.). The addition of 16 minims of water, and boiling, with quick chilling, makes a fresh, sterile 1-1000 suprarenalin solution. It should be boiled in alkali free glass or in porcelain.

The addition of two or more minims of adrenalin to each hundred of anesthetic emphasizes the need of a balanced solution. Without adrenalin the body fluids would quickly absorb and equalize the pressure; and, unless the solution contained irritants, there probably would be no serious annoyance. The vaso-constrictor action of adrenalin, however, retards absorption, and puts the entire burden on the body cells surrounding the injection field. If this injection be much below or above 0.9 per cent. salt solution, we may expect painful infiltrations, indurations, or suppurations, as an aftermath. The degree of osmotic tolerance differs in individuals to a slight extent. Variations in pressure from 0.7 per cent. to 1 per cent. salt solution would give fairly good results. This allows considerable room for error in making up our solutions. The molecule of novocain must be figured in conjunction with the salt. Novocain is isotonic in solutions of 5.48 per cent. As this strength is rather heavy for our work, a corresponding quantity of an indifferent salt,—as sodium chloride,—must be added to the diluted novocain solution to raise its pressure to normal. The use of freshly distilled water is recommended for the making of solutions. A small still can be used in the office, but boiled tap water will be found quite satisfactory. Its effect on osmotic pressure would not be noticeable, and it could be figured as 0.02 per cent. or less. Our San Francisco water, on a fair average, would show about 205 parts of permanent minerals, in suspension in one million. Water boiled in copper containers or sterilizers, however, should be freshly boiled, and not allowed to stand in the copper vessel any great length of time before using, as copper in extremely minute amounts in the water could cause trouble when injected with the anesthetic. Adami comments on the fact that copper, when inserted in a vessel containing polliwogs,. will give up enough copper in three or four hours to end the movements of the polliwogs; and if it acts in a deleterious manner on such a highly organized form of life, the body cell would also suffer in proportion. A porcelain dish for boiling water will be found quite convenient and satisfactory.

The use of stock solutions containing antiseptics to keep them stable should be discouraged; as also the compound tablets. The latter because they are not accurately balanced, are easily deranged by boiling, and do not lend themselves to the changing of solution strengths without a great deal of trouble. For instance, if your patient is a woman, and adrenalin is contraindicated, it would be difficult to eliminate or diminish that part of the tablet. Adrenalin would be contraindicated in pregnancy, and it is thought that susceptibility to untoward results would be increased during menstruation. Care and minimum dosage should be used in thyroid disturbances; and until definite knowledge to the contrary, high blood pressure should indicate minimum dosage in either sex.

Compresed tablets of novocain and adrenalin also retrograde much more rapidly than plain novocain. One of the compound tablets in common use is composed of 1-3 novocain, 1-8 salt, 1-1500 adrenalin. One tablet is supposed to make a 2 per cent. solution upon the addition of 15 minims of water. Eight of these tablets to 120 minims of water would give us one grain of salt. This in 120 minims would make an 0.8 per cent, salt solution, which is practically normal. To this, however, we must figure 2 2-3 grains of novocain. Figuring novocain in fifths it would give us almost 0.6. This added to 0.8 salt solution would give us about 1.4 per cent. osmotic pressure,—a decidedly hypertonic solution, and one capable of causing post-operative annoyance.

### TECHNIC.

After some years of novocain work I find that I have discarded all the elaborate paraphenalia with which I started, and have reduced my methods to ordinary hospital routine. The use of absolute alcohol, alcohol and glycerine, lysol, ethereal soap, etc., for the sterilization of syringes that would not stand boiling has all been discarded, as have the syringes. The more we resort to boiling water for sterilizing and the less we resort to antiseptics the greater are our opportunities of escaping slips in technic and irritation from contaminated solutions. Record and Luer syringes are used. They are wrapped in a napkin and boiled. The napkin prevents violent bumping in the sterilizer and allows gradual cooling upon removal, as they are allowed to remain covered in the napkin. This is important in syringes of the Record make, as the sudden cooling of the metal parts might cause a fracture of the glass barrel.

The novocain and salt can be secured in separate powder papers of one grain each. One grain of salt to 110 minims of water will make a 0.9 per cent. salt solution. If a 2 per cent. novocain solution is desired, add 150 minims of water to the grain of salt. This makes a 0.5 per cent. salt solution. Pour off all but 100 minims, add 2 grains of novocain, and boil. If any evaporation takes place, add enough sterile water to the minim glass to make an even 100 minims. We now have a 2 per cent. novocain salt solution with the same osmotic pressure as a 0.9 per cent. salt solution. With a sterile pipette add one or two minims of 1-1000 adrenalin from the original container, or freshly sterilized suprarenalin solution. This gives 1-100,000, or 1-50,000, and it is quite sufficient in the average case.

If the anesthesia is to be of more than one hour's duration, 4 minims of adrenalin had better be used; 2.5 grains of novocain in 100 minims of 0.4 per cent. salt solution, with 4 minims of adrenalin will give nearly three and one-half hours anesthesia, using 5 cc. of solution. The 2.5 per cent. solution is made by boiling 5 grains of novocain in 200 minims of 0.4 per cent. salt solution, and adding enough sterile water to bring the solution back to the 200 mark in the graduate, as the boiling will cause the loss of some moisture.

The pipette should have a bulb blown in it to prevent the contents from running back into the nipple. Adrenalin would be easily broken up by the sulfur in the rubber.

The salt solution has given the same result as the Ringer tablets, and is a little simpler to understand in shifting the solution strengths. It is quite understood, of course, that surgical cleanliness will be essential to good results. Any swelling following the use of this solution, and appropriate technic, can safely be laid at the door of poor surgery, sharp bone fragments and excessive trauma.

Blocking the inferior dental nerve some authorities recommend injecting on a level with the upper margin of the dental foramen. This entree into a powerfully built jaw results in a collision with the spinous prolongation, or lingula, which gives attachment to the internal lateral ligament of the mandible. The injection, if made at this point, will not always give a successful anesthesia, and it is sometimes necessary to point the needle away from the bone and go around the obstruction. This means injecting away from the bone, in close proximity to blood vessels. If, however, the needle is inserted at the top of the retromolar triangle, through the temporal muscle on the internal oblique line, it can be kept in contact with, or close to, the bone, with no danger of injecting into vessels. The nerve at this place lies between the internal lateral ligament and the bone, and complete diffusion of the anesthetics takes place in about twenty minutes. If the buccinator muscle is to be incised, a few minims of solution must be deposited under the gums buccally to the lower molars. The buccal nerve is given off from the internal pterygoid, which is a division of the same trunk from which the inferior dental is derived (the inferior maxillary nerve). Inferior dental anesthesia, then, would not block these filaments and they must be reached by infiltration. Extracting posterior lower molars without this additional injection will usually result in some response from the patient. If the bicuspid region of the injected side is to be worked on, the incisive filaments from the other side of the mandible must be blocked, and can be reached in the incisive fossa. The process here is cancellous, and diffusion takes place very nicely.

### ADVANTAGES OF REGIONAL ANESTHESIA.

Regional anesthesia has many advantages over general, especially those of the lipoid solvent class, which not only lower resistance and immunity to infection, but also offer absolutely no protection against shock. A patient anesthetized with ether is in practically the same condition—with the exception of psychic impressions—as a patient rendered speechless and tied hand and foot. He can be put in extreme shock from surgical trauma. This is demonstrated by a microscopic examination of the brain cells of a dog that has been subjected to extreme fatigue or trauma while under ether. If two dogs be anesthetized with ether, and if novocain be used on one to block all nerves leading from a certain region, and both animals are subjected to severe trauma for several hours—a histological examination will show that the brain cells of the dog under ether alone will have the characteristic changes induced by shock, while the dog that had its brain protected by novocain blocking will show not only a normal brain, but, I believe, also less swelling and congestion at the site of traumatic injury. Crile has demonstrated in his work at the Lakeside Hospital in Cleveland that shock, post-operative morbidity, etc., can be prevented by combining novocain blocking with gas and oxygen anesthesia and his regular systematic handling referred to as "anoci." He has reduced the mortality in surgical cases from 4.4 per cent., under ether alone, to 1.8 per cent. under "anoci" procedure. Crile's operative technic begins with the patient's entrance into the hospital, but the part in which we are interested is the operating technic. All structures to be incised are first blocked, or infiltrated with novocain, thus preventing any stimuli from reaching the brain. The novocain crystals are boiled in normal salt solution for ten minutes, on two consecutive days; 0.25 per cent. is the strength used. Quinine and urea hydrochloride in strengths of 0.16 per cent. and 0.5 per cent. is used after the operation, and away from the immediate field, but only when the patient will derive more benefit from several days' freedom from pain, then harm, from annoying indurations persisting after the anesthesia.

The quinine salt injected into the tense, unyielding structure on which we dentists must work, would probably be associated with more harm than good. It lowers the resistance of the structures injected and is intensely irritating —causing indurations and sometimes sloughing. Inflammatory changes and even infection, taking place in a nerve confined as the inferior dental is, in a narrow bony canal, can result in permanent damage on account of the lack of space for swelling and the choking of the blood supply.

In the removal of impacted lower molars, with uncovering and irritation of the main nerve trunk, some cerebral irritation and shock is to be expected, especially under ether. Blocking the inferior dental nerve with novocain in these cases, whether your patient is under ether or not, will give splendid results, and, if severe trauma and jagged edges of bone are avoided, the period of convalescence and repair is materially shortened.—*Pacific Gazette*, March, 1915.

